# Retraction Note: Circ_0002770, acting as a competitive endogenous RNA, promotes proliferation and invasion by targeting miR-331-3p in melanoma

**DOI:** 10.1038/s41419-024-06846-9

**Published:** 2024-06-24

**Authors:** Peng Qian, Liu Linbo, Zhai Xiaomei, Pei Hui

**Affiliations:** 1 Department of Plastic Surgery, The First Affiliated Hospital of Zhengzhou University, No. 1 Jianshedong Road, Zhengzhou, Henan Province 450052 China; 2 Department of Emergency, The First Affiliated Hospital of Zhengzhou University, No. 1 Jianshedong Road, Zhengzhou, Henan Province 450052 China

Retraction Note to: *Cell Death and Disease* 10.1038/s41419-020-2444-x, published online 23 April 2020

The Editors-in-Chief have retracted this article. After publication, concerns were raised regarding high similarity between the images presented in this article and a number of other articles on non-coding RNAs [1–5]. The Editors-in-Chief therefore no longer have confidence in the presented data.

The authors have not responded to any correspondence from the editor or publisher about this retraction.
